# COMFORT COMMUNICATION IN HOME HEALTH: FEASIBILITY, ACCEPTABILITY, AND PRELIMINARY EFFECTIVENESS

**DOI:** 10.1093/geroni/igac059.2966

**Published:** 2022-12-20

**Authors:** Sharon Bigger, Noah Zanville, Elaine Wittenberg, Gail Towsley, Lee Glenn

**Affiliations:** East Tennessee State University, Johnson City, Tennessee, United States; HCA Healthcare, North Carolina Division, Asheville, North Carolina, United States; California State University Los Angeles, Los Angeles, California, United States; University of Utah, Salt Lake City, Utah, United States; East Tennessee State University, Johnson City, Tennessee, United States

## Abstract

Home health is the fastest-growing long-term care setting in the U. S.. However, evidence on effective clinician-patient-family communication in home health is lacking. This prospective, two-arm, pre-post randomized controlled trial aimed to assess feasibility, acceptability, and preliminary effectiveness of the COMFORT (Connect, Options, Meaning-making, Family Caregiver, Openings, Relating, and Team) communication model in home health interprofessional staff (IHHS). IHHS (n = 18) were randomized into two groups: Group 1 (control) (n=10) received seven asynchronous modules, and Group 2 (intervention) (n = 8) received the same modules plus a 2-hour synchronous class with interactive slide presentation and exercises. Measures included completion rates, acceptability ratings, comfort with communication in palliative and end-of-life care (C-COPE), and moral distress in health professionals (MMD-HP). Regardless of group, COMFORT was highly acceptable (>4) to IHHS. COMFORT was positively correlated with improved C-COPE scores (p = 0.037). Moral distress scores did not differ before and after the intervention; however, baseline moral distress scores were found to be higher in IHHS when compared to an academic medical center sample from a previous study. Levels of acceptability of COMFORT were significantly related to clinician levels of considering leaving a job due to moral distress (chi square = 7.6, p = 0.02, Kruskal-Wallis rank sum test). Findings suggest that COMFORT training increases IHHS comfort with palliative and end-of-life communication, especially among clinicians with histories of considering leaving a job or having left a job due to moral distress.

